# Blacklisting Health Insurance Premium Defaulters: Is Denial of Medical Care Ethically Justifiable?

**DOI:** 10.1007/s10728-023-00464-w

**Published:** 2023-07-27

**Authors:** Hanna Glaus, Daniel Drewniak, Julian W. März, Nikola Biller-Andorno

**Affiliations:** Institute of Biomedical Ethics and History of Medicine (IBME), University of Zurich, Winterthurerstrasse 30, Zürich, 8006 Switzerland

**Keywords:** Blacklist, Access to health care, Defaulting premium payers, Ethics, Health policy

## Abstract

Rising health insurance costs and the cost of living crisis are likely leading to an increase in unpaid health insurance bills in many countries. In Switzerland, a particularly drastic measure to sanction defaulting insurance payers is employed. Since 2012, Swiss cantons – who have to cover most of the bills of defaulting payers - are allowed by federal law to blacklist them and to restrict their access to medical care to emergencies.

In our paper, we briefly describe blacklisting in the context of the Swiss healthcare system before we examine the ethical issues involved in light of what is known about its social and health impacts. We found no evidence that blacklisting serves as an effective way of recovering unpaid health insurance contributions or of strengthening solidarity within the health insurance system. Furthermore, the ambiguous definitions of what constitutes an emergency treatment and the incompatibility of the denial of medical care with the obligation to provide professional assistance complicate the implementation of blacklists and expose care providers to enormous pressure.

Therefore, we conclude that blacklists and the (partial) denial of medical care not only pose profound ethical problems but are also unsuitable for fulfilling the purpose for which they were introduced.

## Introduction

Switzerland has a universal health care system; purchasing basic health insurance from one of the currently around 60 health insurance providers is compulsory for all persons residing in Switzerland. Volume and pricing of basic health insurance packages are federally regulated. The premiums are set per capita and vary by age, but not by income. Minors are not covered by their parents’ health insurance, but by individual health insurance contracts at the expense of their parents. Over one forth of residents, however, receive subsidies from their canton of residency to cover premiums. Subsidies depend on income thresholds, which vary by canton. Mandatory health insurance expenses are covered by municipalities or cantons for social assistance beneficiaries and recipients of supplementary old-age and disability benefits but not for undocumented immigrants [32]. Documented migrants have a right to social security benefits, but this can result in the non-issuance of a permanent residence permit and bar access to Swiss citizenship, which creates a strong incentive not to claim social security benefits. Premium subsidies are paid to the insured and not to the health insurers, meaning that persons who would be entitled to such subsidies, but do not claim them, are still liable for the full insurance premium towards their insurers. This can pose significant problems since a significant number of blacklisted persons would be entitled to premium subsidies, but does not claim them due to a lack of knowledge or other administrative hurdles [29].

With the introduction of Article 64a into the Swiss Federal Health Insurance Act in 2012, the federal government of Switzerland authorized cantons to blacklist insured persons who do not pay their health insurance contributions. For people on the blacklist, health insurers are only required to cover emergency treatment. All other medical services are postponed until the debt is paid. In the meantime, the health insurance company initiates debt collection against the failing premium payer. Collection proceeds in the usual way (repayment of the debt, legal proposal or garnishment) until a certificate of loss is issued in the event of insolvency. In the event of a certificate of loss, the canton assumes 85% of the unpaid debt.

The number of individuals not paying their health insurance contributions has increased in recent years: from 140’054 in 2016 to 174’071 in 2019 [7]. Of these 174,071 people with unpaid health insurance contributions, the Federal Office of Public Health (Bundesamt für Gesundheit [BAG]) counts 33’159 persons with postponed healthcare interventions, in 2019 [7], including 856 children and adolescents up to the age of 17 [2]. Currently, five cantons (Thurgau, Zug, Lucern, Aargau, Ticino) blacklist failing premium payers and deny them non-emergency medical care. One of these cantons (Zug) is currently in the process of abolishing its blacklist [9]. The canton of Thurgau even had a blacklist for minors whose insurance premiums were unpaid, but has abolished it as of January 2021 [8]. Four cantons (Solothurn, Graubünden, Schaffhausen, Saint Gall) had introduced blacklists but have abandoned them. Although there are political controversies in Switzerland around blacklists, some cantons consider the model a success and would like to continue [33]. In December 2021, the Swiss Parliament has voted (with a very slim majority) to keep the blacklisting system [27].

There are currently no studies which provide detailed insights on the reasons why persons fail to pay their health insurance premiums and get blacklisted. In an analysis of demographic data from the blacklists of the cantons of Zug, Schaffhausen (abolished in 2021) and Thurgau from 2013, Frey and Meier (2015) found that a large majority of persons blacklisted were between 19 and 65 years of age, and only a very small number of blacklisted persons was above 65 years old (as there are special healthcare social security benefits for this group). Roughly two thirds of defaulting insurance payers were male. 60% of persons defaulting on health insurance premiums in one year had already defaulted the previous year. Whilst blacklists were primarily introduced to target those unwilling to pay, Frey and Meier (2015) conclude that likely a significant number of persons on blacklists are rather defaulting due to an unability to pay. They assume that many persons on blacklists would actually be entitled to claim social security benefits, but fail to do so, or struggle with financial hardship, but are only slightly above the income threshold for social security benefits [12].

Similar problems with increasing failing premium payers are reported in other countries such as the United States, the Netherlands or Germany. In the Netherlands, failing premium payers are subject to having premiums plus a sanctioning surplus charge deducted from their wages [13, 35]. In Germany, failing premium payers insured by public health insurance can be imposed a 12% surplus charge per year, whilst failing premium payers insured by private health insurance can be switched to an emergency rate (*Notlagentarif*) which only covers emergency healthcare, palliative and acute pain care, and pregnancy and new mother care [11, 36]. In the United States, unpaid debt can lead to a loss of insurance coverage after a 90-day grace period [34].

Until now, research on the Swiss blacklists has mainly focused on their effects on the payment behavior of individuals unable or unwilling to pay their health insurance contributions and on the cost-benefit ratio. In a study commissioned by the canton of Zurich a comparison of unpaid health insurance contributions in cantons with and without blacklists showed no reducing effect of blacklisting on unpaid health insurance contributions. Blacklisting did not improve the payment behavior of failing premium payers and thus had no economic benefit for the canton [12]. Another study investigated the reasons that led to unpaid health insurance contributions (in general, not limited to blacklisted persons). In this study, about half a million insured individuals were analysed for their sociodemographic characteristics. The study showed that 61% of the individuals not paying their health insurance contributions were rather young, single, healthy and rarely received a premium subsidy. The other 39% were elderly, chronically ill or lived in households with relatively high health expenditures [5]. As studies with undocumented migrants from Geneva have shown, limited access to primary care is associated with poorer health [15, 39]. Surprisingly, to our knowledge, the health or social effects of blacklisting on the listed people in Switzerland have not been investigated so far. Therefore, the aim of our paper is to analyse the ethical implications of blacklisting individuals, while also providing some evidence from health care systems outside Switzerland on the medical effects of denied care.

### The Problem of Defining Medical Emergencies

In Switzerland, blacklisted persons are still entitled to emergency care. According to the Swiss federal law on health insurance, emergency treatments are exempt from the denial of medical care. However, the law does not offer a definition of “emergency treatment”.

From a medical and a legal perspective, emergency treatment allows for a broad range of interpretation. In general, an emergency describes a difficult, sudden situation in which help is urgently needed [17]. An emergency can, however, also be more narrowly defined as an “acute, life-threatening clinical condition due to disturbance of vital functions or the risk of sudden, irreversible organ damage due to trauma, acute illness or intoxication” [20]. Case law also includes situations in which serious symptoms (high fever, severe pain) are to be alleviated [37]. In addition, it remains unclear as to whether and to what extent diagnostic procedures – which might help establish or rule out an emergency - are covered for blacklisted individuals.

These unclarities regarding the coverage of blacklisted patients are also problematic for health care professionals, who run the risk of not being reimbursed or having to enter into negotiations with insurance companies. The lack of a clear definition of emergency treatment also leads to physicians assessing cases differently. If the intention of blacklisting - to exert pressure on people to pay their health insurance premiums – is to be kept up, the concept of emergency must be interpreted restrictively. This, however, forces healthcare professionals into difficult situations, not only from a moral or psychological point of view but also regarding professional legal duties to provide assistance in case of urgency even if not life-threatening [3, 16].

## Methods

The purpose of the present article is to provide an ethics analysis of the Swiss blacklisting system on the basis of a review of the published empirical studies, policy documents and media reports on (i) the purpose of the Swiss blacklists; (ii) their effectiveness to achieve this purpose; (iii) their unintended effects; and (iv) strategies to reduce their unintended effects.

Ideally, an ethics review should take place before or while a health policy measure is being introduced. If a measure fails the ethical analysis, it should not be further pursued. The present analysis was conducted eigth years after the introduction of blacklisting into the Swiss health care system and it is possible that the policy will be abandoned again in the next few years. We hope our study can inform health policy-makers in other countries with insurance-based healthcare systems struggling with similar issues.

A strong limitation of our analysis is the overall scarcity of empirical studies examining the social, economic and health care effects of blacklists. More research would be needed to understand, e.g., the indirect health costs of blacklisting, the reason for which persons end up on the blacklists, and the coping of blacklisted persons and their physicians with the blacklisting system. The available evidence is, however, sufficient to ground medical and ethical concerns about the blacklisting system.

## Results

### Goals of the Blacklists as a Public Health Measure

The Swiss cantons that use blacklists intend to reach two goals: (a) to avoid costs that would accumulate for the canton by having to cover the bills of those failing to pay their premiums; (b) to strengthen the compulsory health insurance system built around the idea of solidarity among the insured by encouraging insurance holders to pay their premiums [24, 33]. The threat of restricted access to medical care is intended to improve the payment behavior of all insured persons. The actual denial of medical benefits serves as a tool – in addition to debt collection, which can lead to garnishment of income and assets and, in the event of proven insolvency, the issuance of a forfeiture certificate - to exert pressure on those who are late in paying health insurance premiums. If the measure reached its goal of improving the payment behavior of all insured persons, it would help save money by reducing unpaid health insurance contributions [12]. This would respond to the ethical principle of justice as it would allow health insurance premiums to be lowered for all insured and would reduce “freeriding” by some at the expense of the community of insured.

Politically, blacklists are promoted as a tool targeting those unwilling to pay their insurance premiums rather than those unable to pay. Indeed, theoretically those who cannot afford to pay their premiums should receive subsidies or have their premiums covered by social security, whereas those who have significant social or psychological problems and do not manage to submit their payments for that reason should receive assistance or, in the case of legal incompetence, a guardian who takes care of these matters for them. This is assuming, however, that the system works perfectly or at least almost. The finding that around 40% of blacklisted persons have indeed severe health and social problems, however, casts a strong doubt on the (political) assumption that blacklists primarily affect those unwilling to pay [5].

### Effectiveness of Blacklists in Reaching Stated Goals

The effectiveness of blacklists with a view to the first goal - reducing costs at the taxpayer’s expense – was examined in 2015 in a study by the health department of the canton of Zurich. In this cost-effectiveness study, the initial costs for the introduction of the blacklist were estimated as between CHF 200,000 and CHF 600,000 and for its management as between CHF 850,000 and CHF 1.8 million annually [12]. In addition, the denial of medical coverage can potentially lead to indirect costs (which are, however, difficult to quantify). A lack of primary care leads to more frequent hospital visits and emergency treatment. With emergency consultation remaining the only access to health care while the severity of illness increases, costs are increasing. These costs are borne by the health care providers in the case of a non-emergency consultation. Such inconsistent treatment focused on emergencies rather than on prevention and early detection and management is inefficient and also increases the burden of disease for those affected. Regarding the second goal – strengthening solidarity by ensuring all who benefit contribute their share -, the study found no difference in the rate of unpaid premiums in cantons with a blacklist as compared to those without such a list. This study showed that in the first three years after introduction of a blacklist neither of the two goals – save money and improve payment morale - was achieved.

### Unintended Effects of the Measure

Implementing blacklists can lead to a number of unintended effects that put a burden on the concerned individuals and society as a whole.

- *Blacklisting as stigma*: Blacklisting individuals who are not able or willing to pay their health insurance contributions stigmatizes - whether intended or not - those who are placed on such a list. As of this writing, names of blacklisted individuals are accessible to a comparatively wide circle of health care providers and health insurance companies. This information could be used to the detriment of the listed individual, for instance in the context of a job application [29]. A recent non-representative survey of ten Swiss chief physicians of university hospitals reported that the patients’ status with regards to blacklisting is noted in the patient file [25]. Such a note may lead to bias on the part of the healthcare team and may have a negative impact on the patient-provider relationship. This is illustrated by a US study that showed an association of stigma and low insurance status, which lead to the experience of discrimination, disrespectful behavior, unfounded removal without treatment, unfair treatment and/or failure to recognize or meet needs [1]. Prejudices resulting from the status of the patient in terms of his or her health insurance coverage have therefore been shown to reduce the effectiveness of care. We may assume that the stigma associated with being blacklisted can cause similar effects.
- *Economic disadvantages for providers*: Given the vague legal framework, situations arise in which it is unclear whether the insurance, the health care provider or the patient has to pay for what is deemed to be emergency treatment of a blacklisted patient. In order to avoid a breach of professional duties, providers will be inclined to bear the treatment costs in case of doubt. However, accepting the risk of financial losses through the treatment of blacklisted patients may be becoming more difficult with increasing economic pressure on healthcare institutions to be more cost-efficient or to produce larger margins [28]. This development can be expected to further complicate access to treatment for this patient group. In the worst case, patients will be rejected already on admission before any professional assessment. The restrictions of medical care for blacklisted patients furthermore creates incentives to shift costs to other service providers: If the attending physician prescribes medication instead of handing it over directly, the pharmacist must bear the potential financial loss [25]. Such manoeuvers increase the risk of treatment discontinuation.
- *Blacklisting as a health risk*: The excess health risk of blacklisted patients can arise from two main effects of medical care denial: First, it can be assumed that blacklisted patients experience not only difficulties accessing care but also treatment delays and altogether fewer medical services than other patients. A blacklisted patient is more likely to abstain from treatment because he or she will fear that the insurance company will not provide reimbursement [31]. If treatment is sought and provided, it begins at an advanced stage of the disease, as persons unwilling or unable to pay their health insurance contributions will probably only seek treatment for pressing problems and may not have a primary care physician referring them on time [6, 30]. The fear of stigmatization and the associated feelings of shame increase the inhibition threshold for reaching out for medical help. As studies from the US show, people with a low insurance status are at higher risk of having an advanced tumor stage when diagnosed with cancer [10, 22]. Blacklisting also endangers the health of dependent minors and unborns, who could be endangered by untreated medical conditions of their parents (e.g., psychiatric conditions or gestational diabetes). Moreover, blacklisting can also endanger public health more broadly since it constitues a barrier of access of blacklisted persons to vaccination (e.g., HPV), testing and treatment of infectious diseases (e.g., Hepatitis B and C or HIV) or other conditions which can potentially endanger the health of others (e.g., diseases which increase the potential for traffic accidents, for instance alcohol abuse disorder or even diabetes mellitus or age-related macular degeneration). Second, blacklisted patients receive lower quality care. The uncertainty about reimbursement can motivate the blacklisted patient and/or the healthcare provider to seek out inexpensive diagnostics and therapeutic options and compromise on quality or comfort. As shown by a US systematic review and meta-analysis from 2020, uninsured patients in both the adult and pediatric population had worse trauma outcomes than insured individuals, as measured by odds of death and all-cause mortality [26]. Additionally, the insurance status also influences the amount and intensity of diagnostic imaging that patients receive in the emergency room [19]. Sparse use of diagnostic measures limits a timely differential diagnosis. In clinical situations where a vital threat has to be routinely excluded, it is not clear who will pay for the diagnosis. Supply gaps in the follow-up of emergency treatments of blacklisted patients have also been witnessed [25], which can have a significant impact on medium- or long-term outcomes, for instance when complications are overlooked or no rehabilitative measures are available to restore function [14, 30].
- *Blacklisting particularly affects vulnerable patient groups*: Chronically ill people are at an increased risk of experiencing financial constraints due to loss of earnings [38]. In addition, a chronic illness often leads to an increase in medical expenses [21]. Therefore, it is not surprising that patients with a chronic disease make up for a significant part of the blacklist in Switzerland and suffer particularly from restrictions or even discontinuation of their medical care [5]. In one reported case, this led to the death of an HIV-infected person as a result of the outbreak of AIDS-related diseases after discontinuation of antiretroviral therapy [4]. However, a study from the University Hospital Geneva, which evaluated the processes and outcomes of 198 diabetes type 2 patients, of which 40% were uninsured, did not find any difference in quality of care between insured and uninsured patients [14]. It can, however, be assumed that newly occurring chronic diseases will be diagnosed less frequently while a patient is blacklisted, delaying the start of therapy and increasing the risk of faster disease progression. Even when a chronic disease, such as arterial hypertension of a blacklisted patient who received emergency treatment for a myocardial infarction, is diagnosed, secondary prevention will not be part of the treatment package.
- *Blacklisting undermines standards of good medical practice*: In routine care, health care professionals are expected to discuss therapy options with the patient and weigh them based on the patient’s personal preferences and side effect profiles [23]. The denial of medical coverage can undermine such participatory decision-making. This happens, for example, when the healthcare provider feels compelled to offer only cheaper treatment options or when he or she directly chooses the cheapest treatment option without involving the patient. Also, core medical tasks are not covered for blacklisted patients, such as the prevention of disease and care for the dying. Withholding palliative care from a blacklisted patient seems hard to reconcile with respect for human dignity.
- *Blacklisting conflicts with medical ethos*: The revised Declaration of Geneva issued by the World Medical Association describes the ideal patient-physician relationship as follows: “The health of my patient will be my first consideration”. Physicians are expected to prioritize the interests of their patients over their own interests or those of third parties. Optimal access to comprehensive health care is in the patient’s interest. A physician who wants to maximize his or her chances of being promoted might limit or postpone a blacklisted patient’s treatment out of self-interest in order to achieve his or her department’s economic goals. Blacklisting therefore pitches patients’ interests against those of physicians and puts patients’ trust at risk as well as physicians’ loyality towards their patients. The Code of Medical Ethics of the American Medical Association describes the promotion of continuity in the care of a patient as a medical obligation. If the physician withdraws from the relationship due to the patient’s insolvency, he or she violates his or her duty of loyalty and risks serious harm for the patient, particularly in the case of long-term relationships between chronic patients and persons of trust such as family doctors, psychiatrists or rheumatologists.
  Physicians who are neither willing to compromise with regards to their patients’ care nor to accept a financial loss for themselves or their institution may find themselves tempted to cheat: If physicians declare regular treatment as emergency treatment for billing purposes, they ensure that they will be paid for the interventions they performed. One study asked about the frequency with which physicians manipulated bills in order to ensure reimbursement for the care they considered necessary for their patients. 39% of the surveyed physicians reported that they had deceived the insurer at least once in the previous year [40].

### Strategies to Reduce Negative Effects of Blacklists

Particularly vulnerable patients who are likely to experience health impairment when medical services are withdrawn can be protected from denied treatment by being excluded from the blacklist. The department of Health and Social Affairs of the canton of Aargau wrote a guidance document in February 2019, in which it recommended that municipalities categorically exclude certain patient groups from blacklisting: chronically ill people whose disease, if left untreated, severely limits their quality of life, or leads to death, or patients who are contagious if left untreated as well as pregnant women. Alternatively, (state-funded) contact points for basic services could be created for persons unable or unwilling to pay health insurance contributions and other “uninsured” persons (at the example of contact points for, e.g., undocumented migrants or homeless persons). Outreach services such as school medical care or psychiatric services for people without disease awareness could also protect blacklisted patients from damages to their health [31].

Communication is also key to mitigating negative effects. In the case of a new list entry, the case management of the canton of Thurgau approaches directly the failing premium payers and informs them of the impending denial of medical services. If the person concerned violated the obligation to pay the premium due to financial difficulties, the municipality contacts him or her and advises, if necessary, to ask for debt counselling [12].

## Discussion

Blacklists and the denial of medical coverage are reprehensible from both ethical and economic perspectives. Blacklisted individuals are severely restricted in their access to health care. There is some evidence that they are treated less frequently, less timely and with interventions of less value, which puts them at higher risk of health impairments than paying insurance holders. Furthermore, the vague definition of what constitutes emergency treatment and the incompatibility of the denial of medical care with the obligation to provide professional assistance complicate the implementation of blacklists and expose care providers to ethical dilemmas. To date, relatively few studies are available that provide empirical evidence of these effects. It is mainly individual cases of adverse health effects from blacklisting that have been documented.

The economic goal of the blacklist is to reduce unpaid health insurance contributions and thereby save costs for the state. The moral goal is to educate people to pay their premiums and thus to contribute duly to a universal healthcare system based on solidarity. The fact that unpaid health insurance contributions are increasing in spite of the existence of blacklists casts doubts on how effectively these goals have been reached if at all. The empirical findings raise the question of why blacklists and the denial of medical care has not improved the payment behavior of insured persons. One reason might be that the debt enforcement process, which is enacted when premiums are not paid, already forces people to settle up unpaid health insurance contributions, even without the additional pressure resulting from the denial of medical care so that blacklists make no difference. In some Swiss cantons, the cantonal legal basis allows to blacklist demonstrably insolvent persons. In the case of insolvency, the denial of medical care is ineffective because those individuals literally cannot pay.

Another reason could be that a considerable number of chronically ill people struggle to pay their fees although they receive subsidies and/or social assistance [5]. Denying this vulnerable group of people access to treatment will impose an additional hardship on them without making a difference for the rate of paid premiums. If people with financial difficulties are denied access to primary care, their social situation may worsen: health limitations reduce quality of life and have a potentially negative impact on employment and financial situation. This is particularly problematic given that the denial of medical care disproportionately affects vulnerable individuals, such as the chronically ill and those experiencing poverty. Thus, blacklisting threatens the health of populations that already have higher mortality and morbidity due to social factors. Health limitations, as well as belonging to low socio-economic positions, reduce a person’s life chances. If denial of medical care leads to an increased combined occurrence of these conditions, it worsens equality of opportunity within society.

The doubtful benefits of the blacklist seem out of proportion with the potentially severe disadvantages for those affected, health care providers and the patient-physican relationship. The blacklist destabilizes the principle of social health insurance, which prohibits exclusion. By creating a way to circumvent compulsory insurance, which is particularly attractive to low-income individuals, the blacklist leads to a reduction in insurance coverage for the economically weakest.

Against this background, we suggest to examine the following approaches to abolish blacklists and to introduce a system which encourages solvent insured persons to pay their premiums. The majority of solvent failing premium payers are young, healthy adults without claim to reduction of health insurance contribution. Precisely this group of people is only marginally affected by denial of medical care and should – instead of being excluded – be encouraged to take personal responsibility through a case management approach, meaning that, e.g., local social workers would establish dialogue, advice persons on existing social security benefits they could claim, and help in negotiations with health insurers in cases of (temporary) inability to pay. Education and debt counseling can help individuals managing their budget and prioritize their expenses [18]. In case of refusal to pay, despite pressure from the current enforcement procedure, the automatic seizure of part of the income for the payment of premiums could be an appropriate way. In cases where subsidy recipients are not capable of managing them properly (e.g., due to substance use disorder), subsidies should be paid directly to the health insurance company.

## Outlook

Our article has shown ground for serious ethical concerns about the blacklisting system. However, further empirical work would be helpful to better grasp the actual impact of the denial of medical care on the health of blacklisted individuals. Especially the perspective of affected patients and their caregivers is not sufficiently represented in current research. Motivation for refusal to pay and implications for privacy and the patient-physicians relationship could be better outlined with qualitative research. Whether the doubts about the legitimacy of blacklists can be the basis for a lawsuit before a higher court would be a question that could be clarified in further legal investigations. A comparative analysis between countries facing the same challenge might yield interesting opportunities for learning and improvement.

## Data Availability

Not applicable.
